# Performance Evaluation of Structural Health Monitoring Anomaly Data Processing Algorithms in Resource-Constrained Edge Computing

**DOI:** 10.3390/s26144658

**Published:** 2026-07-22

**Authors:** Kuanjiu Lei, Yaojie Li, Shitong Hou, Wenlong Yan, Yongjun Qin, Feiyu Zuo

**Affiliations:** 1 School of Architecture and Engineering, Xinjiang University, Urumqi 830017, China; 107556523308@stu.xju.edu.cn (K.L.); qyjjg@xju.edu.cn (Y.Q.); 2 School of Civil Engineering, Southeast University, Nanjing 210096, China; 220231527@seu.edu.cn (Y.L.); shitonghou@seu.edu.cn (S.H.); 3 National and Local Joint Engineering Research Center for Intelligent Construction and Maintenance, Nanjing 210096, China; feiyuz265@163.com

**Keywords:** edge computing, structural health monitoring (SHM), anomaly data processing, machine learning, performance evaluation

## Abstract

Edge computing plays an important role in structural health monitoring (SHM) for transportation infrastructure because it enables local data processing and low-latency decision support. However, SHM systems encounter challenges due to data anomalies caused by sensor faults, transmission errors, or irregular structural behavior. This study evaluates the deployment performance of a SHM anomaly data-processing workflow on resource-constrained edge devices. The workflow includes data cleaning, response separation, and anomaly detection. Missing data are processed using cubic spline interpolation. Jump points and drift are corrected using the Laida criterion, and noise is reduced using wavelet threshold denoising. Response separation is then performed using the detrending method based on time windows, the 3σ criterion, or wavelet packet decomposition. Anomaly detection is then performed using autoregressive integrated moving average with explanatory variables, support vector machines, and recurrent neural networks. Simulation data and field monitoring data from bridge displacement and highway pavement strain are used to evaluate the workflow. The evaluation focuses on runtime, memory usage, central processing unit usage, and a data-processing throughput proxy. This analysis helps to understand the performance and trade-offs of algorithms on edge devices under the resource constraints typical of SHM applications.

## 1. Introduction

Transportation infrastructure, such as roads and bridges, is the cornerstone of urban life and economic development. With the advancement of sensing technology, structural health monitoring (SHM) has become essential for ensuring the long-term stability of these infrastructures [1]. SHM detects structural damage or degradation using sensing technology and structural feature analysis [2]. Currently, there are two main approaches to structural state assessment and damage detection using SHM data: model-based and data-driven methods. Model-based methods rely on physical or mechanical models of structural behavior. In contrast, data-driven methods mainly use the statistical and temporal characteristics of monitoring data, without requiring detailed physical modeling of the structure. However, SHM systems inevitably generate monitoring data that includes anomalies caused by sensor faults, transmission errors, or structural behavior irregularities. Identifying these anomalies and determining their causes is crucial for appropriate intervention [3,4].

With the development of Internet of Things (IoT) technology, wireless sensor networks have become a core component of SHM systems, providing real-time monitoring and generating massive amounts of data [5]. A typical wireless sensor network consists of sensors and communication modules. These data are transferred to the cloud for analysis and processing [6]. However, this centralized cloud architecture can introduce latency and performance issues, especially for devices located far from the data center [7]. Edge computing offers a new solution. Unlike traditional cloud computing, edge computing processes data on edge devices [7,8]. This approach allows certain data-processing tasks to be executed locally under a fixed resource budget, reducing the need to send and receive data from the cloud [9]. Therefore, some anomaly data-processing algorithms can be embedded in edge devices to preprocess monitoring data and detect anomalies in real-time, reducing the burden on data transmission. Recent edge-intelligence studies have also emphasized that model deployment on resource-constrained devices is influenced by model size, latency requirements, and edge–cloud collaboration. Mao et al. [10] proposed a multilayer mobile edge-computing framework with knowledge distillation to balance detection accuracy and response latency. Although their study focused on fall detection rather than SHM, it provided useful evidence that model deployment and latency–resource trade-offs are critical in edge-intelligence systems.

In the field of SHM, data preprocessing, which encompasses data cleaning and response separation, is essential for ensuring high-quality data and enhancing the reliability of SHM analysis. Data cleaning typically addresses distortions, such as missing values, outliers, drift, and noise [11]. To address missing or Not-a-Number (NaN) data in time series, Luo et al. [12] used least-squares regression to impute missing monitoring data from steel structures. Hou et al. [13] proposed a deep-learning and data-augmentation framework for data imputation when multiple sensors fail. For outliers and baseline drift, the Laida criterion has been used in engineering data-cleaning practice [14]. Yi et al. [15] developed a Bayesian robust tensor learning model to reconstruct monitoring data with outliers, drift, and noise. Wavelet-based denoising methods are prevalent in image processing, speech processing, and SHM [16,17]. Additionally, response separation is crucial for identifying and distinguishing the effects of various factors on the structure, such as those induced by temperature and vehicles. This separation is vital for understanding structural behavior and conducting effective anomaly detection. In the separation of strain or displacement data, the detrending method based on time windows and the 3σ criterion is commonly used to estimate baseline trends and extract vehicle-induced responses. Zhao et al. [18] applied wavelet packet decomposition to extract vehicle-induced responses and isolate them from temperature-induced responses. Furthermore, machine-learning methods are increasingly used for anomaly detection because SHM systems generate large volumes of time-series data. These methods range from conventional statistical regression models to more advanced machine-learning and deep-learning methods, such as support vector machines (SVMs) and deep neural networks [19]. For example, Nicolas et al. [20] employed various machine-learning models, such as autoregressive integrated moving averages with explanatory variables (ARIMAX), SVMs, and recurrent neural networks (RNNs), to detect anomalies in bridge displacement monitoring data. Ni et al. [21] proposed a deep-learning framework for anomaly detection, data compression, and reconstruction in long-term bridge monitoring.

Although many data-preprocessing and anomaly-detection algorithms have been developed for SHM, most existing studies focus primarily on methodological innovation or detection accuracy under offline computing conditions. The practical deployability, resource consumption, and hardware–algorithm matching of these algorithms on resource-constrained edge devices remain insufficiently investigated. Therefore, this study does not aim to propose a new individual algorithm. Instead, it provides a deployment-oriented benchmarking evaluation of a complete SHM anomaly data-processing workflow on lightweight industrial edge devices.

The main contribution of this study is to integrate established data-cleaning, response-separation, and anomaly-detection methods into a unified edge-computing workflow. The workflow is then evaluated on two lightweight edge platforms under consistent benchmarking conditions. The evaluation indicators include runtime, memory usage [22], central processing unit (CPU) usage [23], and data-processing throughput proxy [24]. This provides practical evidence for selecting appropriate algorithms and edge hardware in SHM applications with limited computing resources. Specifically, the data-cleaning algorithm employs cubic spline interpolation for missing data, the Laida criterion for outlier and drift processing, and the wavelet threshold denoising method for noise reduction. The response-separation algorithm uses the detrending method based on time windows, the 3σ criterion, and wavelet packet decomposition for the corresponding processing and comparative analysis. The anomaly-detection stage compares ARIMAX, SVM, and RNN models.

The structure of this paper is as follows: Section 2 details the hardware configuration and embedded algorithms of edge devices; Section 3 presents the algorithm debugging and testing using simulation data; Section 4 evaluates the workflow using field monitoring data from bridge displacement and highway pavement strain; and Section 5 summarizes the main findings, limitations, and future research directions.

## 2. SHM Anomaly Data-Processing Based on Embedded Computing Platforms

### 2.1. Compute Box Hardware Configuration

In recent years, the development of IoT and edge computing has driven the widespread adoption of Advanced RISC Machine (ARM)-based lightweight industrial edge-computing devices [25]. The Rockchip RK3588 and RV1126 processors, manufactured by Rockchip Electronics Co., Ltd. in Fuzhou, China, exemplify high-performance, low-power ARM architecture chips, making them particularly well-suited for edge-computing applications. In this study, these two processors were selected to represent lightweight edge-computing platforms with different resource levels. They were used to evaluate the feasibility of real-time SHM data processing on edge devices. These two processors were selected because they represent two typical levels of lightweight industrial edge-computing platforms used in engineering monitoring applications. RK3588 represents a relatively high-performance ARM-based edge platform, with stronger computing capability, larger memory capacity, GPU/NPU support, and better Linux software compatibility. In contrast, RV1126 represents a low-power and more resource-constrained embedded platform. It is suitable for lightweight preprocessing tasks, but it has limited support for computationally intensive machine-learning models. Therefore, the comparison between RK3588 and RV1126 enables an engineering-oriented evaluation of how the same SHM anomaly data-processing workflow performs under different resource constraints. As shown in Table 1, the Rockchip RK3588 is a highly equipped processor featuring a powerful multi-core architecture and an advanced graphics processing unit. It supports multiple neural-network-computing precisions, high-resolution video processing, several interface options, and both Android and Linux operating systems. These features make it suitable for high-performance edge applications. In contrast, the Rockchip RV1126 is a low-power processor with fewer cores and more limited computing capability. It does not include a dedicated graphics processing unit. Python and relevant scientific computing libraries were installed on the Linux operating systems of both devices to enable on-device algorithm deployment and execution. However, several large-scale numerical and deep-learning libraries could not be successfully installed on the RV1126 platform because of architectural compatibility constraints and limited system resources. As a result, the five anomaly-detection models, which rely on these libraries for numerical computation and matrix operations, could not be executed on RV1126. They were therefore evaluated only on RK3588. All computations in this study were strictly executed on the edge devices, and no external computation or cloud offloading was used.

### 2.2. Embedded Algorithms

**Figure 1:**
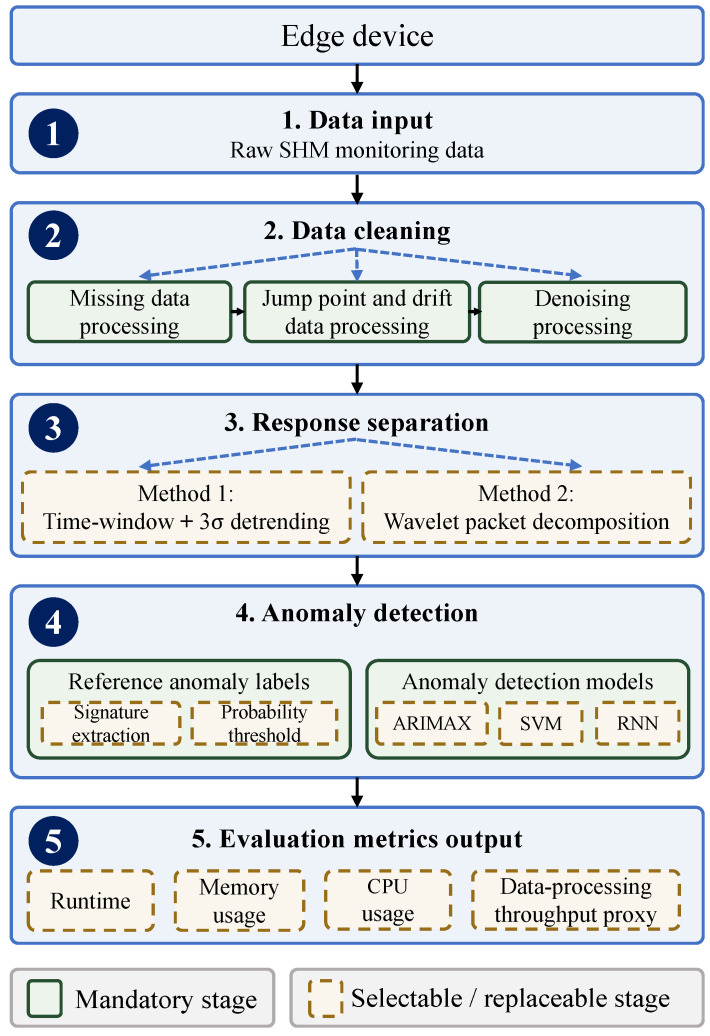
Flow chart of the embedded SHM anomaly data-processing workflow on edge devices.

The overall flowchart of the embedded SHM anomaly data-processing workflow on edge devices is shown in Figure 1. Preprocessing and cleaning of raw monitoring data consists of three steps: identification and processing of missing values, addressing jump-points and drift data, and denoising. These steps in turn clean the raw data to ensure the integrity and reliability of the raw data. In the response-separation stage, two different methods, namely the detrending method based on time windows and the 3σ criterion and wavelet packet decomposition, are used to further process the cleaned data, effectively separating the temperature-induced response data from the vehicle-induced response data. In the anomaly-detection stage, ARIMAX, SVM, and RNN models are employed to process the vehicle-induced response data and identify data anomalies.

#### 2.2.1. Data-Cleaning Algorithm

**Figure 2:**
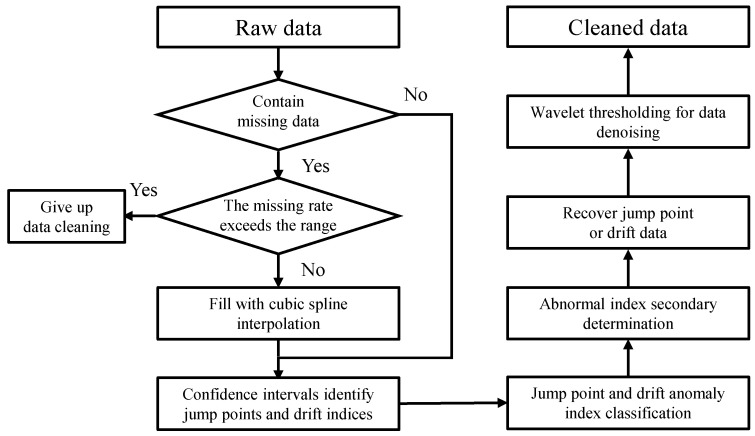
Flow chart of the data-cleaning algorithm.

The data-cleaning algorithm employed in this study addresses issues related to missing data, data jump points, drift, and noise, as illustrated in Figure 2. For handling missing data, the algorithm first assesses the original dataset to identify constant and missing values, subsequently filling the missing positions using cubic spline interpolation. The procedure for addressing data jump points and drift comprises the following steps: (1) calculating the difference between the original data and a reference value, generating a difference series, and performing a confidence analysis to establish the threshold range for normal data, thereby preliminary identifying outlier indices; (2) classifying the anomaly index characteristics associated with jump points and drift based on the initial anomaly index sequence; and (3) reassessing the drift index list, considering the original data characteristics to mitigate the impact of pseudo-jump points and avoid erroneous cleaning. Finally, the identified anomaly data for jump points is normalized, and the drift segments are corrected according to the local baseline trend. For noise data, wavelet threshold denoising is applied using the sym3 wavelet basis function, a three-layer wavelet decomposition, the VisuShrink universal threshold calculation rule [26], and the soft thresholding method.

#### 2.2.2. Response-Separation Algorithm

In SHM, deflection and strain responses are typical time-series data affected by both low-frequency environmental effects and dynamic excitation. Temperature variation usually introduces slowly varying baseline trends into monitoring data. Vehicle movement or transient loading generates dynamic response components. Therefore, the temperature-induced response component needs to be removed from the raw data so that the vehicle-induced response component can be extracted more accurately. This system employs two methods to process the data to achieve this effect.

Method 1 is the time-window and 3σ-based detrending method. The monitoring data are first divided into fixed time windows. The window size is determined according to the sampling frequency of the data. Within each window, a threshold is established using the 3σ criterion. The average value of the data points within this threshold is then calculated and used as the baseline value of the window. This baseline represents the temperature-induced response trend. The vehicle-induced response is obtained by subtracting this trend from the original data.

Method 2 is wavelet packet decomposition. This method decomposes the measured response signal into different frequency bands. The temperature-induced response is mainly located in the low-frequency band, whereas the vehicle-induced response is mainly located in the high-frequency band because of its transient characteristics [15,18]. Therefore, vehicle-induced response data can be obtained from the signal portion of the high-frequency band. Discrete signals can be decomposed into different frequency bands step by step using discrete wavelet packet transformation. The decomposition scale of the wavelet packet transform can be determined by the spectrum of the signal. It is assumed that the frequency of the temperature-induced strain data is mainly at [0, *f*_t_]. According to sampling theory, for a signal with sampling frequency *f*_r_, the available frequency band is [0, *f*_r_/2]. The zeroth decomposition sequence of the *N*-layer using the wavelet packet transformation is at [0, *f*_r_/2*^N^*^+1^]. The optimal decomposition scale of a wavelet packet transformation packet can be determined by choosing a suitable *N*, such that *f*_r_/2*^N^*^+1^ is slightly greater than *f*_t_ and *f*_r_/2*^N^*^+2^ is less than *f*_t_. In the datasets evaluated in this study, the Daubechies 6 mother wavelet was selected because it provides a suitable balance between smoothness and compact support. Furthermore, based on *f*_r_ of the structural responses, the optimal decomposition level was explicitly determined to be *N* = 9. It can satisfy the *f*_t_ conditions for isolating the environmental baseline.

#### 2.2.3. Anomaly-Detection Algorithm

The anomaly-detection stage evaluates three types of time-series models: ARIMAX, SVM, and RNN. The evaluation focuses on assessing the predictive performance of these models in edge-computing environments and the computational efficiency of edge devices. Two SVM models are employed: one with a linear kernel (denoted as SVM_lin_) and the other with a radial basis function kernel (denoted as SVM_RBF_). The RNN employs both long short-term memory (LSTM) and gated recurrent unit (GRU) models.

The anomaly labels used in this study are binary reference labels, where zero represents normal data and one represents anomalous data. These labels were generated within the proposed processing workflow based on the data obtained after data cleaning and response separation. Therefore, they should be interpreted as workflow-generated reference labels rather than independently verified ground-truth labels. For regression-based models, including ARIMAX and support vector regression (SVR), residuals between observed and predicted values were compared with a 3σ-based threshold to assign anomaly labels. For LSTM and GRU, the binary reference labels generated from the processed data were used as training targets, and a probability threshold of 0.5 was applied for anomaly classification. This formulation allows different models to be evaluated under the same preprocessing and reference-labeling framework. However, the evaluation mainly reflects model behavior and computational feasibility under a consistent workflow rather than classification performance against independently verified ground truth.

The ARIMAX model extends the traditional ARIMA model by incorporating additional exogenous variables, enhancing its ability to capture external factors influencing time-series data. Maximum likelihood estimation and cross-validation methods are used to estimate the parameters of the ARIMAX model. The linear model expression for m time-series observations x(t) using ARIMAX is shown below [27]:

(1) $${∆}^{d}y\left(t\right)=\overset{p}{\underset{i=1}{\sum }}\propto_{i}{∆}^{d}y\left(t-i\right)+\overset{q}{\underset{j=1}{\sum }}\theta_{j}\varepsilon \left(t-j\right)+\overset{m}{\underset{k=1}{\sum }}\beta_{k}x_{k}\left(t\right)+\varepsilon \left(t\right)$$

where $\propto_{i}$ and $\theta_{j}$ are the parameters of the autoregressive and moving average processes, respectively; ${∆}^{d}$ represents the dth-order difference; and $\varepsilon \left(t\right)$ denotes the residual of $y\left(t\right)$. For anomaly detection in time-series monitoring data using ARIMAX, anomalies are identified based on the residual error. If the residual error exceeds three times the standard deviation, the point is considered an anomaly.

The SVR model applies SVM techniques to regression problems. SVR maps data from a low-dimensional space to a high-dimensional feature space using a kernel function. This allows nonlinear regression problems to be solved through linear regression in the transformed feature space [28]. This paper utilizes regression models based on both linear and radial basis kernel functions. The expressions for the linear and radial basis kernel functions are as follows:

(2) $$K_{lin}\left(x_{i},x_{j}\right)={x_{i}}^{T}\cdot x_{j}$$

(3) $$K_{rbf}\left(x_{i},x_{j}\right)=\exp\left(-\gamma {∥x_{i}-x_{j}∥}^{2}\right)$$

where $K_{lin}\left(x_{i},x_{j}\right)$ denotes the linear kernel function, and $K_{rbf}\left(x_{i},x_{j}\right)$ represents the radial basis kernel function. The variables $x_{i}$ and $x_{j}$ are the two input vectors, and γ is the parameter that controls the width of the kernel. For anomaly detection in time-series monitoring data using the SVR model, the residual of the test data is first calculated. Then, the root mean square error (RMSE) of the residual is multiplied by a threshold factor to determine the anomaly threshold. The threshold factor is typically set to three, and any data point with a residual exceeding this threshold is considered an anomaly.

LSTM is a type of RNN that uses gating mechanisms to control the forgetting, updating, and output of information. Its basic structure includes a forget gate, an input gate, and an output gate. The forget gate regulates the amount of information discarded from the current cell state [29]. In this study, the LSTM model has a two-layer network structure, with 150 units in each layer. A dropout layer with a dropout rate of 0.5 is used to reduce overfitting. This structure improves the ability of the model to represent sequential data and learn dependencies in complex feature sequences. For anomaly detection, the predicted probability is compared with a threshold of 0.5. A data point is considered anomalous when the predicted probability exceeds this threshold.

GRU is a simplified version of LSTM. It uses update gates and reset gates to control information updating and forgetting. In GRU, the cell state and hidden state are merged into a single hidden state [30]. In this study, the GRU model also has a two-layer structure, with 150 GRUs in each layer. A dropout layer with a dropout rate of 0.5 is used to reduce overfitting. Because GRU has fewer parameters than LSTM, it may provide a more stable training process. For anomaly detection, the predicted probability is compared with a threshold of 0.5. A data point is considered anomalous when the predicted probability exceeds this threshold.

### 2.3. Evaluation Indices of Edge Computing

To evaluate the performance of these anomaly data-processing algorithms on resource-constrained edge devices, four metrics are selected: runtime, memory usage, CPU usage, and data-processing throughput proxy. All the code in this paper is implemented using the Python programming environment. Runtime is measured using the time module. By recording the start and end times before and after executing the algorithm, we can determine the execution time of the algorithm. This metric assesses the speed and efficiency of the algorithm. For the machine-learning models, the reported runtime refers only to the inference stage and does not include model training, hyperparameter tuning, data loading, or file I/O. Memory usage is monitored with the psutil library, which measures the resident set size of the process, representing the amount of memory occupied by the process in random access memory. By comparing memory usage before and after algorithm execution, we quantify the additional memory consumption during execution. This metric is crucial for evaluating the memory efficiency of the algorithm, particularly on resource-constrained devices. CPU usage is also tracked using the psutil library. This method calculates CPU utilization over a specified time interval. By comparing CPU usage before and after executing the algorithm, we measure the change in CPU utilization due to the algorithm. This approach provides a detailed analysis of the computational load and helps understand the efficiency and performance of the function under varying operational conditions. The fourth indicator was defined as a data-processing throughput proxy rather than a standard hardware-level measurement of floating-point operations per second. The tested edge platforms did not provide reliable access to low-level hardware performance counters. Therefore, the actual number of floating-point operations during algorithm execution could not be directly measured. In this study, the data-processing throughput proxy was calculated as the processed data volume divided by runtime under the same data format and benchmarking protocol. This indicator was used only for relative comparison of data-processing throughput among different algorithms on the same hardware platform. It should not be interpreted as a direct measurement of floating-point operations per second or as an indicator of theoretical computational complexity.

To ensure a reproducible benchmarking protocol for these metrics, we implemented a strictly controlled experimental environment. All benchmarks were conducted on a clean operating system (Ubuntu 20.04 for RK3588 and Buildroot Linux 2024.02 for RV1126), with all non-essential background services terminated to minimize system noise. Both platforms were configured with a standardized Python 3.8 environment. To reduce runtime variation caused by dynamic frequency scaling and multi-threading interference, the CPU governor was locked in performance mode, and the execution was restricted to a single CPU core. Each data segment was processed 10 times to compute an average, and the results from 10 different segments were further averaged to obtain the final performance metrics. For memory usage, the initial runs serve as a warm-up phase for memory allocation. Thus, the reported memory usage represents a stable comparative delta for evaluating relative resource intensity, rather than an absolute peak footprint. Therefore, the memory results are suitable for comparing the relative memory intensity of different algorithms under the same benchmarking protocol. Continuous peak memory profiling and explicit memory headroom analysis should be included in future deployment-oriented evaluations. Furthermore, the system cache was strictly cleared prior to processing each segment to prevent residual states from influencing the results. Under these controlled conditions, the reported averages provide a stable basis for comparing the relative deployment performance of the algorithms on the tested edge devices.

## 3. Simulation Tests and Results

### 3.1. Overview of Simulation Tests

Simulation tests were conducted to compare the deployment performance of different algorithms under controlled conditions. The generated random numbers are used in simulation tests to evaluate the performance and computational efficiency of various algorithms on edge-computing devices. Because edge devices have limited computing and storage resources, processing each data point individually may cause excessive resource consumption. Therefore, batch processing was used to improve resource efficiency [31]. In this study, the sample size for each batch-processing test ranged from 1000 to 10,000, with increments of 1000.

As shown in Table 2, different simulated conditions were designed for the data-cleaning algorithms. The evaluated edge-computing performance indicators included runtime, CPU usage, memory usage, and data-processing throughput proxy. For missing data processing, missing rates of 2.5%, 5.0%, and 7.5% were used. For jump-point and drift processing, anomaly rates of 0.5%, 1.0%, and 1.5% were used. For denoising, uniform random noise was added to the simulated data. To calculate the runtime, CPU usage, and data-processing throughput proxy under consistent conditions, the same data segment was processed 10 times, and the average value was recorded. The results from 10 different data segments with the same data volume were then averaged to obtain the final values. For memory usage, the initial runs were treated as a warm-up phase for memory allocation. Therefore, the memory result was calculated from 10 different data segments with the same data volume. The system cache was cleared before each processing run to reduce the influence of residual system states.

### 3.2. Simulation Test Analysis

#### 3.2.1. Data-Cleaning Algorithm

**Figure 3:**
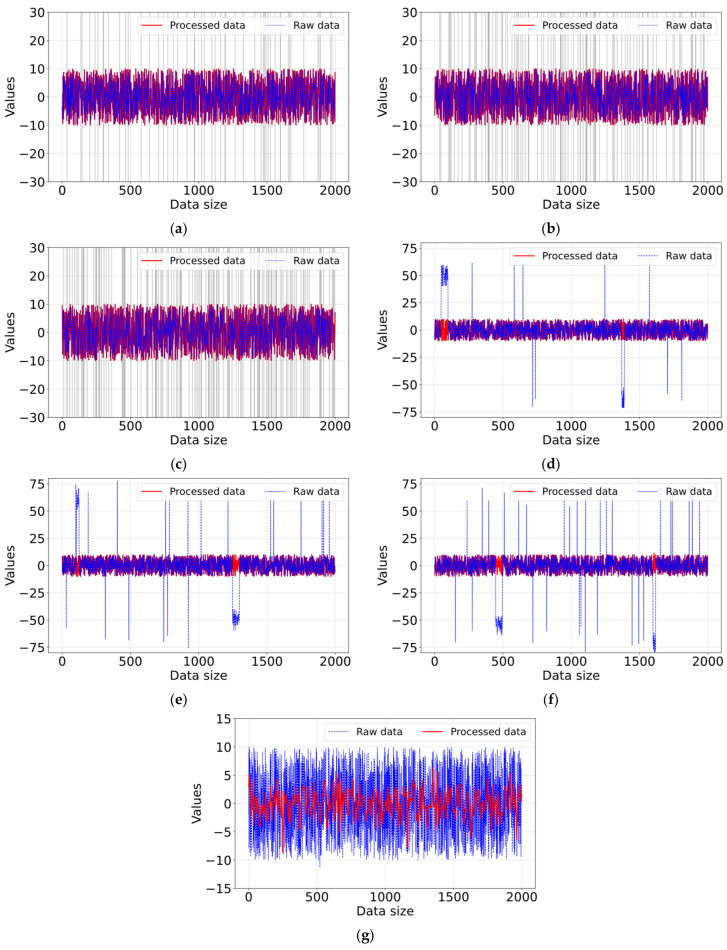
Data-cleaning results under different simulated conditions: (**a**) 2.5% missing rate; (**b**) 5.0% missing rate; (**c**) 7.5% missing rate; (**d**) 0.5% anomaly rate; (**e**) 1.0% anomaly rate; (**f**) 1.5% anomaly rate; and (**g**) uniform random noise.

Figure 3 shows the data-cleaning results under different simulated conditions, using 2000 samples as an example. Figure 3a, Figure 3b, and Figure 3c show the missing data-processing results at missing rates of 2.5%, 5.0%, and 7.5%, respectively. The shaded gray areas in these figures indicate the random deletion positions in the raw data. Figure 3d, Figure 3e, and Figure 3f show the jump-point and drift processing results at anomaly rates of 0.5%, 1.0%, and 1.5%, respectively. Figure 3g presents the performance of the denoising algorithm under the uniform random noise condition. These results demonstrate that the data-cleaning algorithm used in this study effectively preprocesses and cleans the data under simulated conditions.

**Figure 4:**
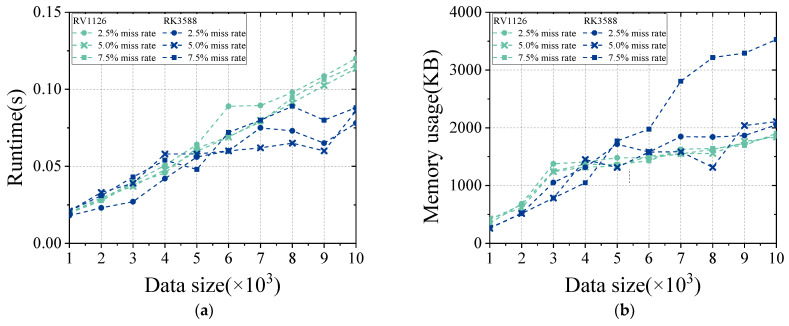

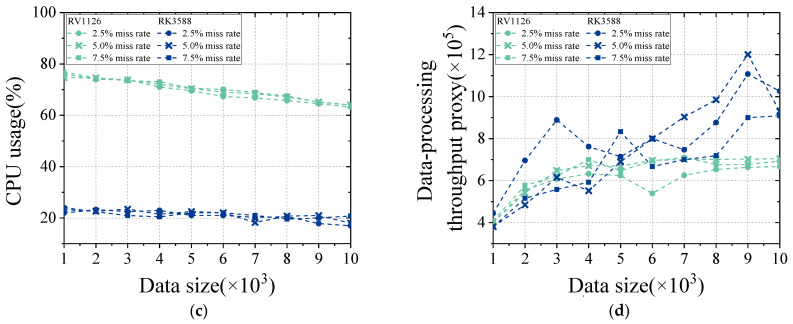
Missing processing algorithm edge-computing index results: (**a**) runtime; (**b**) memory usage; (**c**) CPU usage; and (**d**) data-processing throughput proxy.

Figure 4 presents the results of edge-computing indices for the missing processing algorithm under different data volumes (ranging from 1000 to 10,000 samples) and missing rates (2.5%, 5.0%, 7.5%). The evaluated indicators included runtime, memory usage, CPU usage, and data-processing throughput proxy. With the increase in the data volume and missing rates, the runtime of RK3588 gradually increased from 0.02 s to 0.09 s, while RV1126 gradually increased from 0.02 s to 0.12 s. The memory usage of both RK3588 and RV1126 generally increased with data size. The CPU utilization of the RK3588 fluctuated between 14% and 26%, while that of the RV1126 between 65% and 76%. This may be related to the polynomial calculations required for cubic spline interpolation. Lower missing rates generally resulted in higher data-processing throughput proxy values. An increase in data size also led to an increase in the throughput proxy value, although the relationship was not strictly linear. Under the same benchmarking conditions, the data-processing throughput proxy shows that RK3588 achieved higher relative processing throughput than RV1126, especially as data size increased. Some performance indicators, such as runtime and memory usage, occasionally showed lower values for larger datasets than for smaller datasets. This phenomenon may be attributed to hardware load fluctuations. However, the overall trend increased as data size grew. In general, data size and missing rate affected the performance indicators, with higher resource consumption observed under higher missing rates.

**Figure 5:**
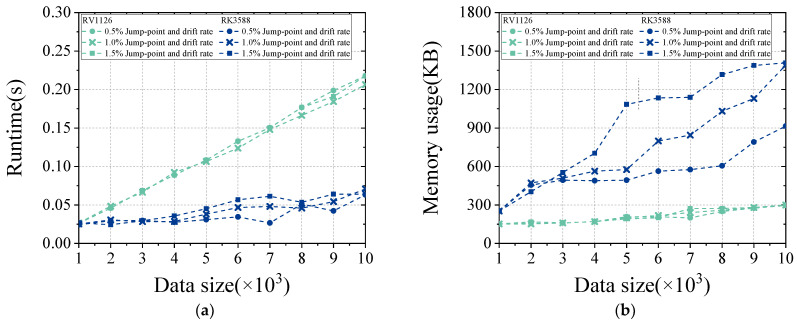

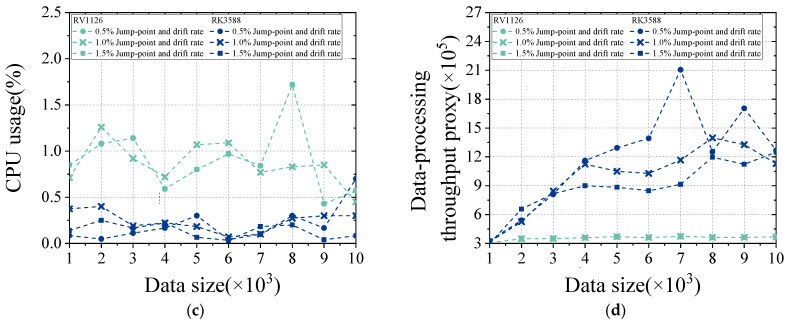
Jump and drift processing algorithm edge-computing index results: (**a**) runtime; (**b**) memory usage; (**c**) CPU usage; and (**d**) data-processing throughput proxy.

Figure 5 presents the edge-computing index results for jump-point and drift processing algorithms under different data volumes and jump-point and drift anomaly rates (0.5%, 1.0%, and 1.5%). Runtime, memory usage, and data-processing throughput proxy all increase with the data size and jump-point and drift anomaly rates, but the specific change trends fluctuate. With the increase in data size and jump-point and drift anomaly rates, the runtime of RK3588 gradually increased from 0.02 s to 0.07 s, while that of RV1126 gradually increased from 0.02 s to 0.23 s. Memory usage increased with data size on RK3588, whereas RV1126 maintained relatively low and stable memory usage. A higher anomaly rate, such as 1.5%, increased memory usage more clearly at larger data scales. CPU usage fluctuated between 0% and 0.6% on RK3588 and between 0.4% and 1.8% on RV1126. No clear correlation was observed between CPU usage and data volume. The data-processing throughput proxy increased with data size, although the relationship was not linear. Under the same benchmarking conditions, RK3588 generally achieved higher relative processing throughput than RV1126.

**Figure 6:**
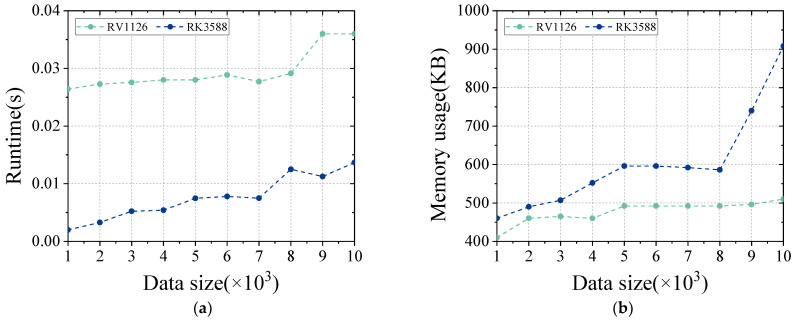

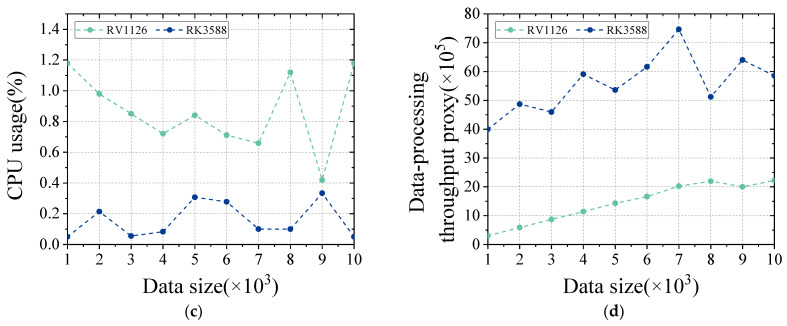
Denoising algorithm edge-computing index results: (**a**) runtime; (**b**) memory usage; (**c**) CPU usage; and (**d**) data-processing throughput proxy.

Figure 6 presents the results of the edge-computing indices for the denoise processing algorithm with varying data volumes. As the volume of data increased, the runtime of RK3588 gradually increased from 0.0025 s to 0.014 s, while that of RV1126 gradually increased from 0.026 s to 0.036 s. The memory usage increased significantly with data size for RK3588, while RV1126 maintained a relatively low and stable memory usage. The CPU usage of RK3588 fluctuated between 0% and 0.35%, while that of RV1126 fluctuated between 0.4% and 1.2%, with no significant correlation between the value and the volume of data. The data-processing throughput proxy also exhibited a fluctuating trend for RK3588, while RV1126 increased steadily. The data-processing throughput proxy indicated that RK3588 generally achieved higher relative processing throughput compared to RV1126.

#### 3.2.2. Response-Separation Algorithm

**Figure 7:**
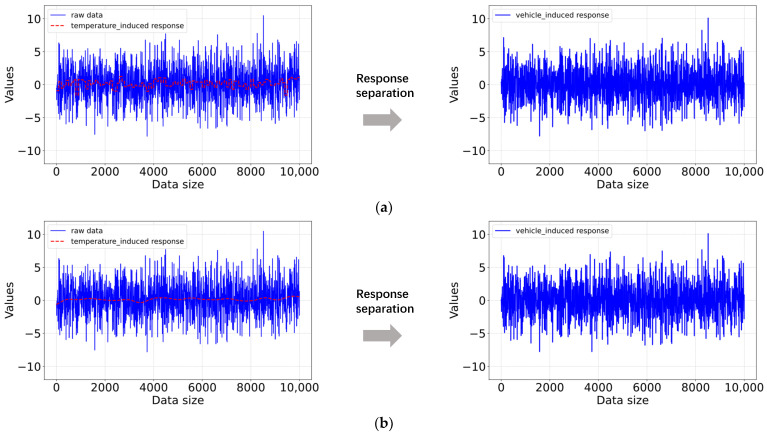
Response-separation algorithm results: (**a**) method 1; and (**b**) method 2.

For the response-separation algorithms, the sample size for each batch-processing test ranged from 1000 to 10,000, with increments of 1000. Figure 7 illustrates the effectiveness of response-separation algorithms by using 10,000 sample data points as an example. Figure 7a and Figure 7b depict the operational outcomes of method 1 and method 2, respectively. The results indicate that both methods can separate response components under simulated conditions.

**Figure 8:**
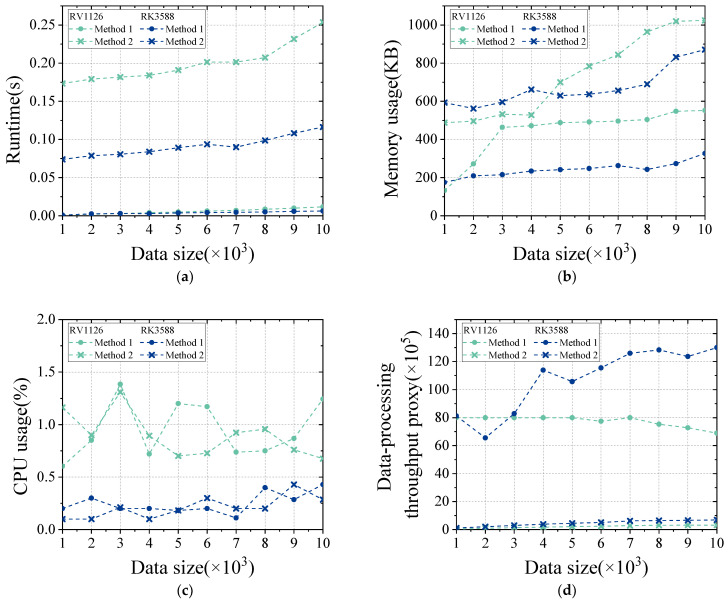
Response-separation algorithm edge-computing index results: (**a**) runtime; (**b**) memory usage; (**c**) CPU usage; and (**d**) data-processing throughput proxy.

Figure 8 shows the edge-computing index results of the two response-separation algorithms under different sample data volumes, including runtime, memory usage, CPU usage and data-processing throughput proxy. For runtime, method 1 remained below 0.01 s on both processors. In contrast, the runtime of method 2 increased with data volume. On RK3588, it increased from 0.08 s to 0.12 s. On RV1126, it increased from 0.17 s to 0.26 s. Method 2 required higher memory usage on both processors, while method 1 required relatively low memory usage. CPU usage fluctuated between 0.1% and 0.5% on RK3588 and between 0.6% and 1.4% on RV1126. No clear relationship was observed between CPU usage and data volume. Under the same benchmarking conditions, RK3588 showed higher data-processing throughput proxy values than RV1126. Method 1 also showed higher throughput proxy values than method 2 on both processors.

#### 3.2.3. Anomaly-Detection Algorithm

**Figure 9:**
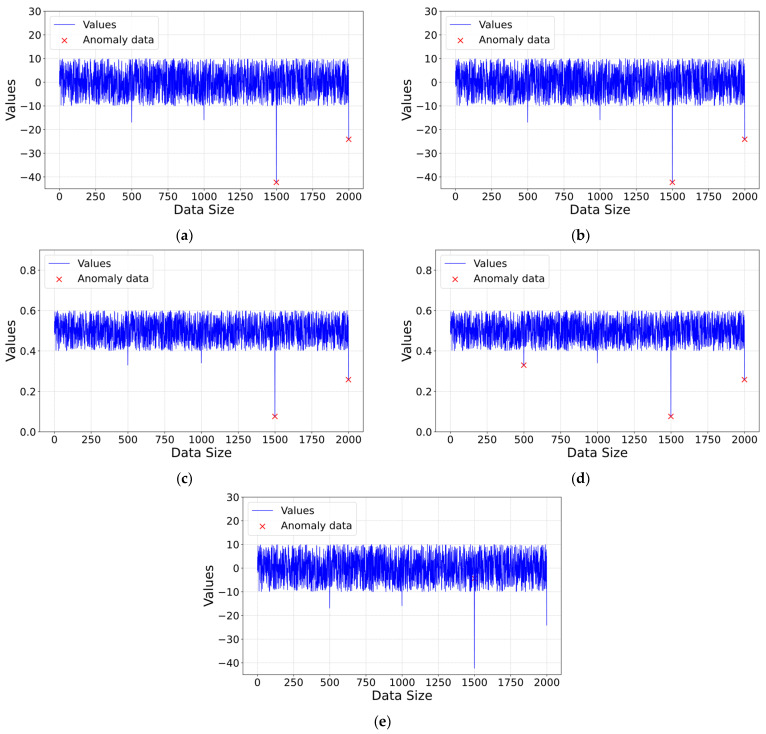
Anomaly-detection algorithm results: (**a**) SVMRBF; (**b**) SVMlin; (**c**) LSTM; (**d**) GRU; and (**e**) ARIMAX.

**Figure 10:**
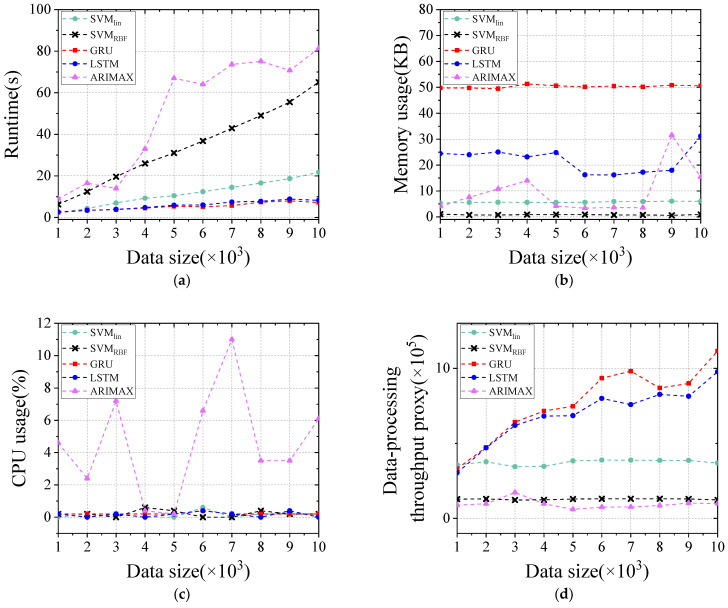
Anomaly-detection algorithm edge-computing index results: (**a**) runtime; (**b**) memory usage; (**c**) CPU usage; and (**d**) data-processing throughput proxy.

After testing, only RK3588 was capable of training and running the aforementioned five machine-learning models. Therefore, in this section, only RK3588 was evaluated based on edge-computing indices. This section set the sample size for a single batch-processing test to range from 1000 to 10,000, with increments of 1000. Figure 9 illustrates the anomaly-detection performance of the five models. Figure 10 presents the edge-computing performance results of each model under different data volumes. In the comparative analysis of various data volumes and algorithm performance, GRU and LSTM exhibited strong performance on large datasets and maintain fast processing speeds. SVM_lin_ operated efficiently with small datasets but increased in runtime as the dataset size grew. Both ARIMAX and SVM_RBF_ required more time and slowed down with increasing data volumes. The CPU usage of GRU, LSTM, SVM_lin_, and SVM_RBF_ remained relatively stable, while ARIMAX showed significant fluctuations. In terms of memory usage, SVM_RBF_ performed best, remaining below 10 MB, followed by SVM_lin_. ARIMAX showed considerable fluctuations in memory usage. LSTM and GRU had higher memory footprints, with GRU often exceeding 50 MB and LSTM fluctuating between 15 MB and 30 MB. As data size increased, the throughput proxy values of GRU and LSTM increased markedly, whereas those of SVM_lin_ and SVM_RBF_ remained low and stable. ARIMAX had the lowest throughput proxy value.

ARIMAX was implemented as a time-series regression model with residual-based anomaly identification, while SVM, LSTM, and GRU were implemented according to their corresponding regression or sequence-learning procedures. Therefore, comparison between these models mainly reflects their computational burden and edge-device deployability under their practical implementation forms. It does not represent a fully identical statistical training protocol. Under this implementation, ARIMAX showed relatively high runtime and larger CPU fluctuation, especially as data volume increased. LSTM and GRU showed better scalability in batch-processing scenarios, but they required more memory. SVM_lin_ was efficient for small- and medium-sized datasets, while SVM_RBF_ may became more time-consuming as data size increased. Therefore, the selection of anomaly-detection models should consider both computational resources and deployment requirements.

## 4. Experimental Tests and Results

### 4.1. Overview of Experimental Tests

To evaluate the effectiveness of the anomaly data-processing algorithms under field monitoring conditions and to assess their deployment performance on edge devices, this paper selected two field monitoring experiments. The first experiment monitored displacement data from the Nanjing Jurong River Bridge in China using a photoelectric deflection meter. The second experiment recorded strain data from sensors installed on the G2 Highway pavement in China. For the field tests, the sample size for each batch-processing test ranged from 1000 to 10,000, with increments of 1000. The runtime, CPU usage, memory usage, and data-processing throughput proxy were then calculated as the edge-computing performance indicators. Table 3 lists the experimental conditions for each algorithm.

### 4.2. Experimental Test Analysis

#### 4.2.1. Data-Cleaning Algorithm

**Figure 11:**
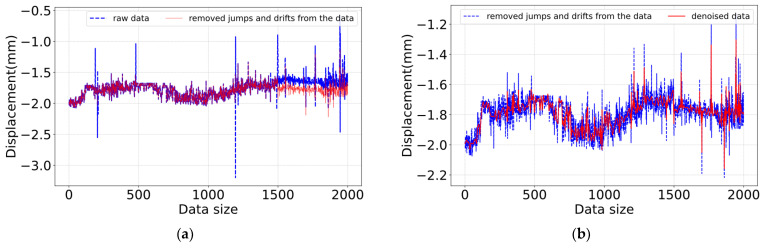
Cleaning results of Jurong River Bridge displacement monitoring data: (**a**) jump-point and drift processing; and (**b**) denoising.

Figure 11 shows the cleaning and preprocessing results for the Jurong River Bridge displacement monitoring data, using 2000 samples as an example. Figure 11a compares the raw data with the data after jump-point and drift processing. Figure 11b compares the data after jump-point and drift processing with the denoised data. The results show that the data-cleaning algorithm can preprocess the bridge displacement monitoring data under field conditions.

**Figure 12:**
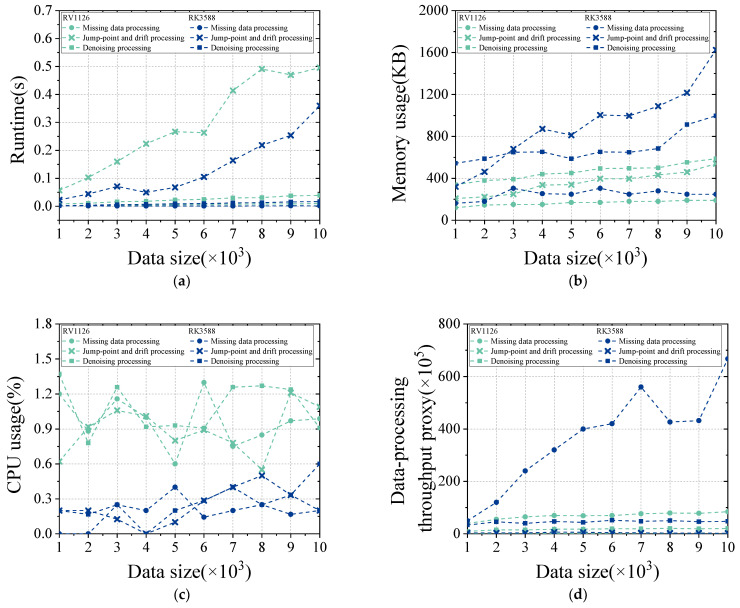
Edge-computing index results of data-cleaning algorithm for Jurong River Bridge displacement monitoring data: (**a**) runtime; (**b**) memory usage; (**c**) CPU usage; and (**d**) data-processing throughput proxy.

Figure 12 shows the edge-computing index results of missing data-processing, jump-point and drift processing, and denoise processing algorithms under different data volumes. The evaluated indicators include runtime, CPU usage, memory usage, and data-processing throughput proxy. As the sample size increased, the runtime of the missing data-processing algorithm on both edge devices remained below 0.01 s. In contrast, the runtime of denoising gradually increased, reaching 0.025 s on RK3588 and 0.05 s on RV1126. The runtime of jump-point and drift processing showed a more obvious upward trend. When the sample size reached 10,000, the runtime was close to 0.4 s on RK3588 and increased to approximately 0.5 s on RV1126. The CPU usage of the three algorithms fluctuated between 0% and 0.60% on RK3588 and between 0.6% and 1.40% on RV1126. No clear correlation was observed between CPU usage and data volume. Under the three algorithms, the memory usage of RK3588 is higher than that on RV1126. The data-processing throughput proxy for missing data processing increased significantly with data scale due to the absence of missing data in the field test set. Denoising showed the second-highest throughput proxy values, while jump-point and drift processing showed lower values. Under the same benchmarking conditions, RK3588 generally achieved higher relative processing throughput than RV1126.

**Figure 13:**
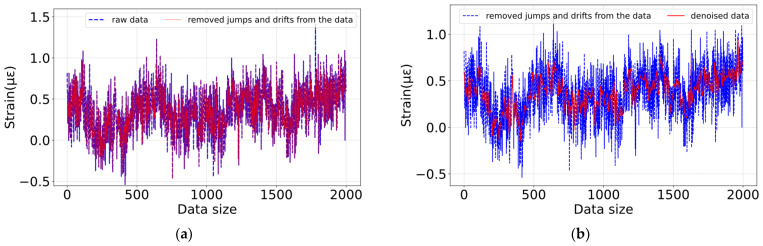
Cleaning results for G2 Highway pavement strain monitoring data: (**a**) jump-point and drift processing; and (**b**) denoising.

Figure 13 shows the cleaning and preprocessing results for the G2 Highway pavement strain monitoring data, using 2000 samples as an example. Figure 13a compares the raw data with the data after jump-point and drift processing. Figure 13b compares the data after jump-point and drift processing with the denoised data. The results show that the data-cleaning algorithm can preprocess the highway pavement strain monitoring data under field conditions.

**Figure 14:**
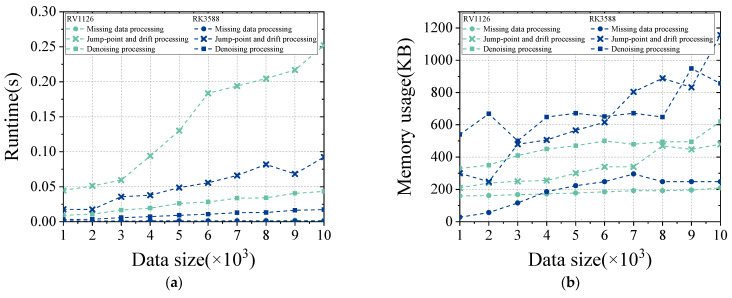

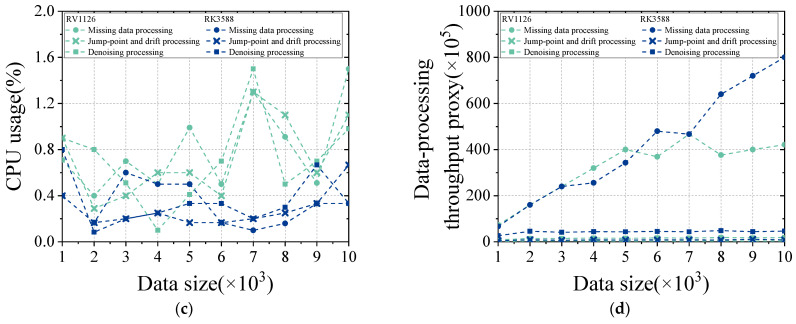
Edge-computing performance results of the data-cleaning algorithm for G2 Highway pavement strain monitoring data: (**a**) runtime; (**b**) memory usage; (**c**) CPU usage; and (**d**) data-processing throughput proxy.

Figure 14 presents the edge-computing performance results of the data-cleaning algorithms for the G2 Highway pavement strain monitoring data. The evaluated algorithms include missing data processing, jump-point and drift processing, and denoising. The evaluated indicators include the runtime, CPU usage, memory usage, and data-processing throughput proxy. As the volume of sample data increases, the running time of the missing processing algorithm of the two edge devices remained below 0.01 s. In contrast, the runtime of denoising gradually increased, reaching 0.02 s on RK3588 and 0.05 s on RV1126. However, the runtime of jump-point and drift processing showed a significant upward trend. When the sample data size reached 10,000, the runtime of RK3588 was close to 0.1 s, while that of RV1126 increased significantly to 0.25 s. The CPU usage of the three algorithms fluctuated between 0% and 0.80% on RK3588 and between 0.2% and 1.50% on RV1126. No clear correlation was observed between CPU usage and data volume. Memory usage increased with data size on RK3588, whereas RV1126 maintained relatively stable memory usage. The data-processing throughput proxy value for missing data processing increased markedly with data size because no missing values were present in the field test set. Denoising showed the second-highest throughput proxy values, while jump-point and drift processing showed lower values. Under the same benchmarking conditions, RK3588 generally achieved higher relative processing throughput than RV1126.

#### 4.2.2. Response-Separation Algorithm

**Figure 15:**
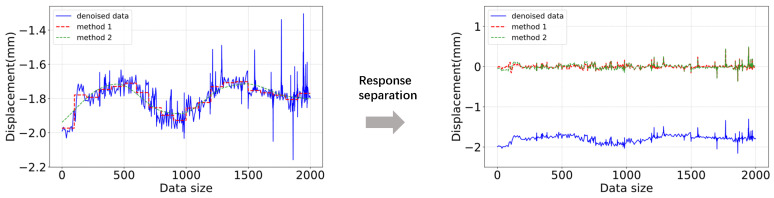
Response-separation effect of Jurong River Bridge displacement monitoring data.

Figure 15 illustrates the effectiveness of the response-separation algorithms, using 2000 sample data points as an example. The results show the performance of method 1 and method 2. The results show that both methods can separate response components in the bridge displacement monitoring data.

**Figure 16:**
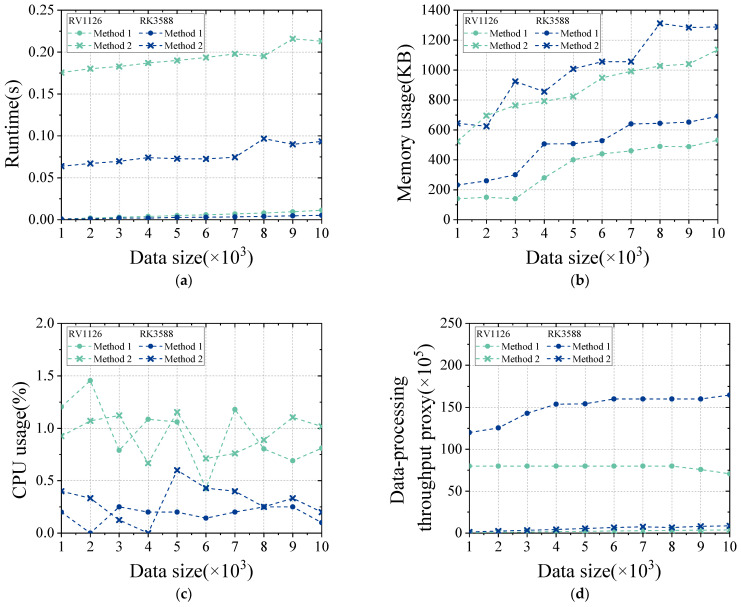
Edge-computing index results of response-separation algorithm for Jurong River Bridge displacement monitoring data: (**a**) runtime; (**b**) memory usage; (**c**) CPU usage; and (**d**) data-processing throughput proxy.

Figure 16 presents the edge-computing index results for the two response-separation algorithms for the Jurong River Bridge displacement monitoring data under different sample sizes. The evaluated indicators include the runtime, memory usage, CPU usage, and data-processing throughput proxy. For both devices, the runtime of method 1 remained below 0.01 s. In contrast, the runtime of method 2 increased with sample size, from 0.06 s to 0.10 s on RK3588 and from 0.175 s to 0.22 s on RV1126. RK3588 showed higher memory usage than RV1126 when both methods were used. The CPU usage of the two methods fluctuated between 0% and 0.60% on RK3588 and between 0.5% and 1.40% on RV1126. No clear correlation was observed between CPU usage and data volume. In terms of the data-processing throughput proxy, method 1 showed higher throughput proxy values than method 2. Under the same benchmarking conditions, RK3588 generally achieved higher relative processing throughput than RV1126.

**Figure 17:**
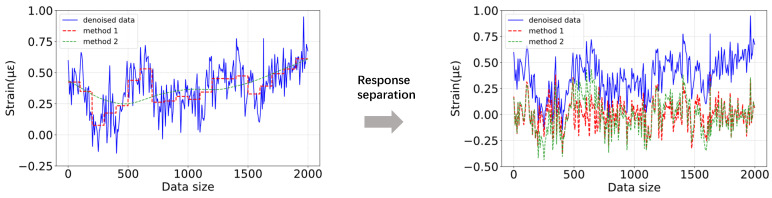
Response-separation results for the G2 Highway pavement strain monitoring data.

Figure 17 illustrates the effectiveness of the response-separation algorithm, using 2000 sample data points as an example. The results show the performance of method 1 and method 2. Both methods were able to separate the response components in the highway pavement strain monitoring data.

**Figure 18:**
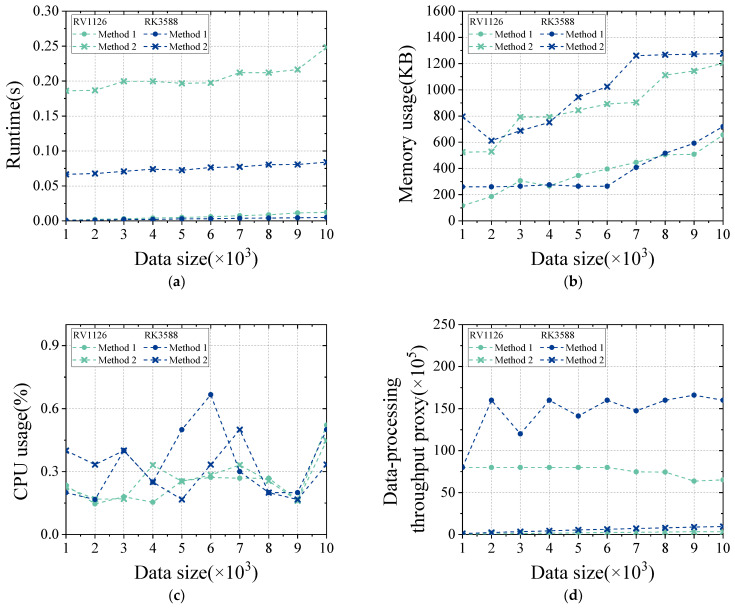
Edge-computing performance results of the response-separation algorithm for the G2 Highway pavement strain monitoring data: (**a**) runtime; (**b**) memory usage; (**c**) CPU usage; and (**d**) data-processing throughput proxy.

Figure 18 presents the edge-computing index results for the two response-separation algorithms for the G2 Highway pavement strain monitoring data under different sample sizes. The evaluated indicators include the runtime, memory usage, CPU usage, and data-processing throughput proxy. For both devices, the runtime of method 1 remained below 0.01 s. In contrast, the runtime of method 2 increased with sample size, from 0.06 s to 0.10 s on RK3588 and from 0.18 s to 0.25 s on RV1126. When method 2 was used, RK3588 showed higher memory usage. Under method 1, memory usage was similar on the two processors. The CPU usage of the two methods fluctuated between 0% and 0.60% on RK3588 and between 0% and 0.5% on RV1126. No clear correlation was observed between CPU usage and data volume. In terms of the data-processing throughput proxy, method 1 showed higher throughput proxy values than method 2. Under the same benchmarking conditions, RK3588 generally achieved higher relative processing throughput than RV1126.

#### 4.2.3. Anomaly-Detection Algorithm

**Figure 19:**
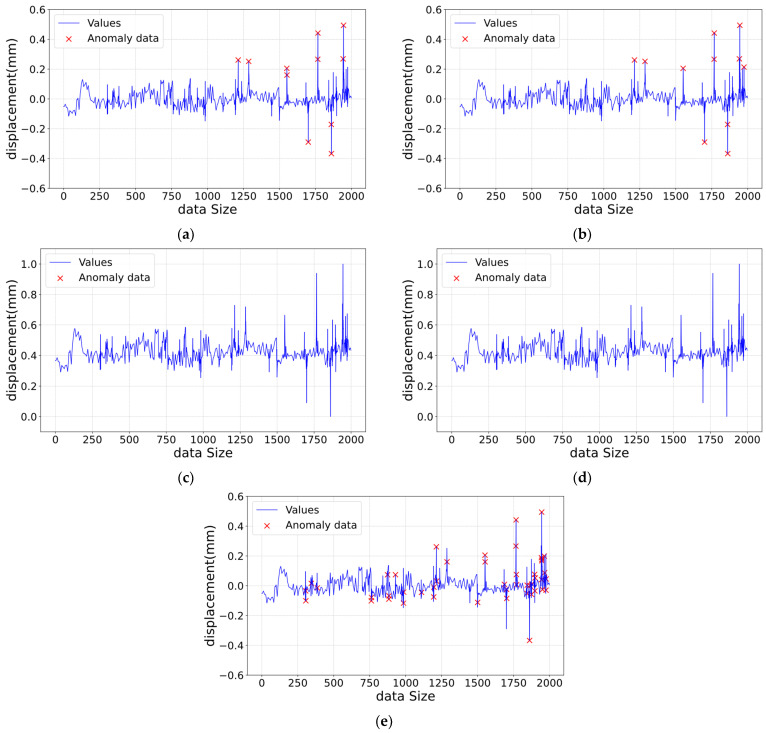
Results of anomaly detection for the Jurong River Bridge displacement monitoring data: (**a**) SVM_RBF_; (**b**) SVM_lin_; (**c**) LSTM; (**d**) GRU; and (**e**) ARIMAX.

**Figure 20:**
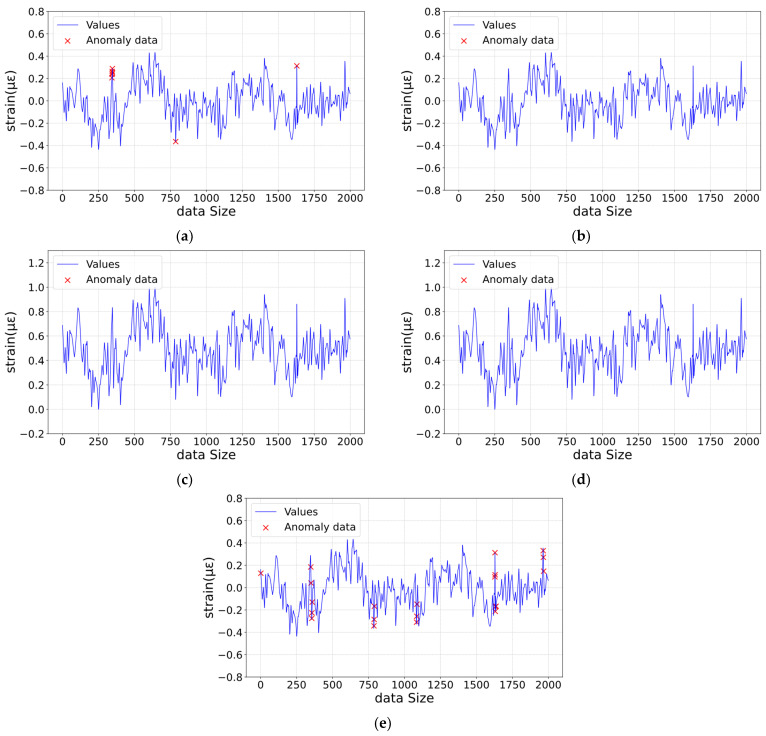
Results of anomaly detection for the G2 Highway pavement strain monitoring data: (**a**) SVMRBF; (**b**) SVMlin; (**c**) LSTM; (**d**) GRU; and (**e**) ARIMAX.

After testing, only RK3588 was capable of training and running the aforementioned five machine-learning models. Therefore, this section evaluates only RK3588 using the edge-computing performance indicators. Figure 19 shows the anomaly-detection results of the five models on the Jurong River Bridge displacement monitoring data. Figure 20 shows the anomaly-detection results of the five models on the G2 Highway pavement strain monitoring data.

To evaluate the prediction behavior of each model, RMSE was used as the error indicator. The average RMSE of the first 30% of the test set was denoted as *μ*_r_. The average RMSE of the remaining 70% of the test set was denoted as *μ*_R_. Model stability was evaluated using the stability coefficient |*μ*_R_−*μ*_r_|. This coefficient describes the difference between early-stage and later-stage prediction errors and reflects the temporal stability of each model.

**Figure 21:**
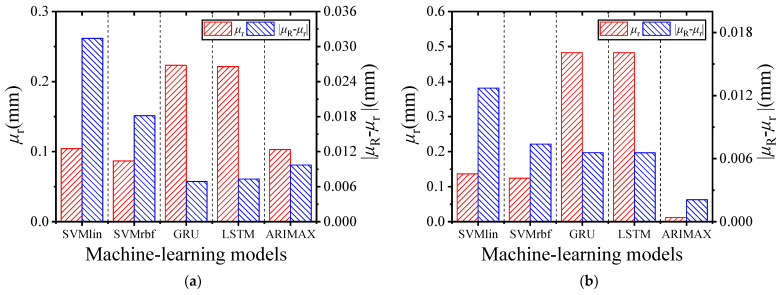
The performance evaluation results of each model in two field scenarios: (**a**) Jurong River Bridge displacement monitoring; and (**b**) G2 Highway pavement strain monitoring.

Figure 21a presents the quantified model performance results for the Jurong River Bridge displacement monitoring dataset. According to *μ*_r_, the SVM_RBF_ model had the smallest error indicator, whereas the LSTM and GRU had relatively larger indicators. According to |*μ*_R_−*μ*_r_|, the LSTM and GRU had the smallest stability coefficients, followed by ARIMAX, while SVM_lin_ had the largest value. Figure 21b presents the quantified model performance results for the G2 Highway pavement strain monitoring dataset. According to *μ*_r_, SVM_RBF_ again had the smallest error indicator, whereas LSTM and GRU had relatively larger error indicators. According to |*μ*_R_−*μ*_r_|, ARIMAX had the smallest stability coefficient, followed by LSTM and GRU, while SVM_lin_ had the largest value. The results in Figure 21a,b show that the models had different levels of prediction error and temporal stability across the two field monitoring scenarios. It should be noted that RMSE and the stability coefficient were used to describe prediction error and temporal stability, rather than complete classification-level detection effectiveness. Because independently verified point-by-point ground-truth anomaly labels were not available for the field monitoring datasets, precision, recall, F1-score, and ROC/AUC were not calculated in this study. Therefore, the results should be interpreted as a comparison of model prediction behavior and edge-computing deployability under the same workflow-generated reference-labeling framework.

**Figure 22:**
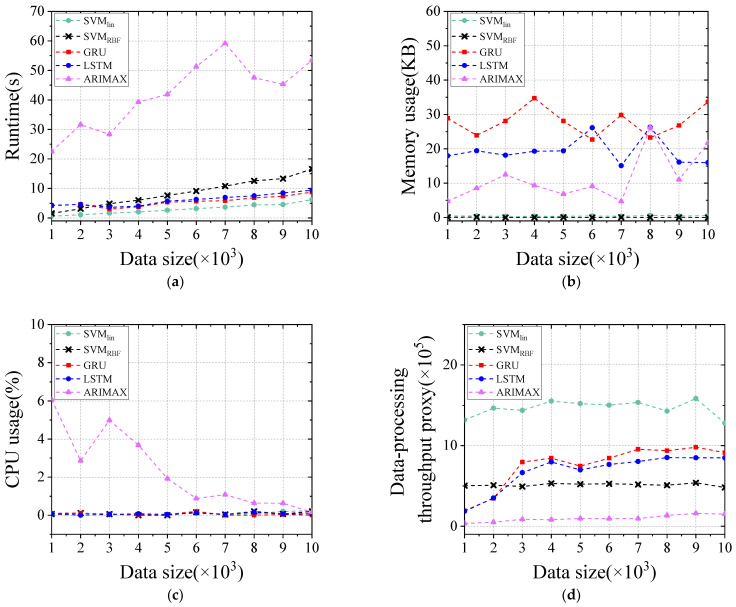
Edge-computing index results of anomaly-detection algorithms on Jurong River Bridge displacement monitoring data: (**a**) runtime; (**b**) memory usage; (**c**) CPU usage; and (**d**) data-processing throughput proxy.

Figure 22 presents the edge-computing performance results of the anomaly-detection models for the Jurong River Bridge displacement monitoring data under different sample sizes. SVM_lin_ and SVM_RBF_ performed well in terms of runtime and memory usage. Their runtimes remained below 20 s, and their memory usage remained below 1 MB. GRU and LSTM exhibited higher memory usage. GRU fluctuated between 20 MB and 40 MB, while LSTM fluctuated between 10 MB and 30 MB. ARIMAX showed less favorable runtime, especially with larger data volumes, where its runtime exceeded 50 s. However, its memory usage was lower than that of GRU and LSTM and generally remained below 20 MB. The data-processing throughput proxy showed that the SVM-based models remained relatively stable as data volume increased, whereas the RNN-based models showed an upward trend. This indicates that RNN-based models may be more suitable for batch-processing scenarios with larger data volumes when sufficient memory resources are available. When considering runtime, CPU usage, and memory usage, SVM_lin_ and SVM_RBF_ are more suitable for real-time processing requirements in this monitoring scenario.

**Figure 23:**
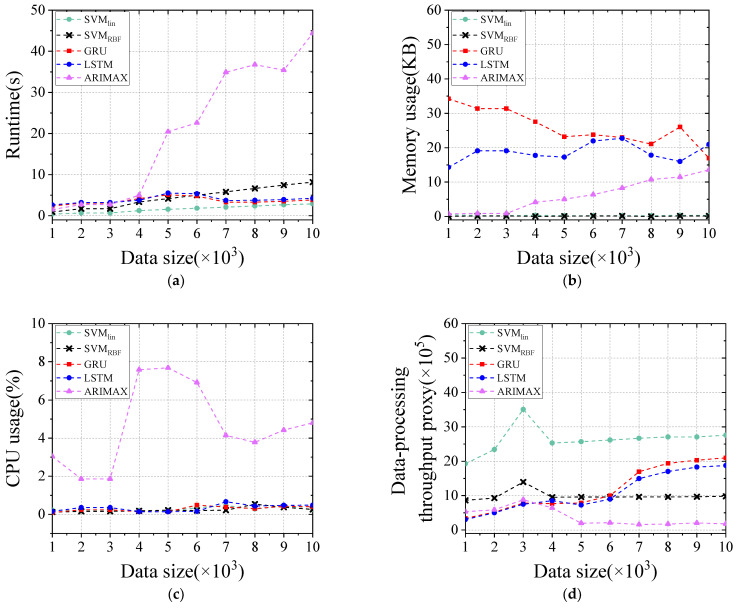
Edge-computing performance results of anomaly-detection algorithms on the G2 Highway pavement strain monitoring data: (**a**) runtime; (**b**) memory usage; (**c**) CPU usage; and (**d**) data-processing throughput proxy.

Figure 23 presents the edge-computing performance results of the anomaly-detection models for the G2 Highway pavement strain monitoring data under different sample sizes. SVM_lin_ and SVM_RBF_ demonstrate efficient performance in terms of runtime and memory usage. Their runtimes remained below 10 s, and their memory usage remained below 1 MB. These characteristics make them suitable for real-time processing of large datasets. GRU and LSTM exhibited higher memory usage, particularly with GRU fluctuating between 20 MB and 40 MB and LSTM between 14 MB and 25 MB. ARIMAX showed less favorable runtime and CPU usage stability, especially with larger data volumes, where its runtime approached 50 s. However, its memory usage was lower than that of GRU and LSTM, and generally remained below 20 MB. When considering runtime, CPU usage, and memory usage, SVM_lin_ and SVM_RBF_ are more suitable for real-time processing requirements in this monitoring scenario. SVM_lin_ performed best in terms of runtime. In contrast, the runtime of SVM_RBF_ increased noticeably with dataset size, as the two SVM variants share the same formulation but use different kernels. Except for ARIMAX, all algorithms showed low CPU usage. In terms of data-processing throughput proxy, SVM_lin_ showed the highest throughput proxy value among the tested models.

### 4.3. Discussion

Because edge devices have limited computing and storage resources, real-time data streams are typically processed in batches. However, the selection of the batch-processing window may cause fluctuations in the performance results, especially for small data volumes. Overall, the observed fluctuations remained within a reasonable range and did not affect the main comparison among algorithms and devices.

For data-cleaning algorithms, the runtime, memory usage, and data-processing throughput proxy generally increased with data volume. This trend was observed for missing data processing, jump-point and drift processing, and denoising. However, CPU usage did not show a clear correlation with data volume. Overall, RK3588 showed better edge-computing performance than RV1126. The runtime of data-cleaning algorithms was generally below 0.5 s, CPU usage was below 1.5%, and memory usage was mostly below 1600 KB.

For response-separation algorithms, method 1 showed lower computational cost, shorter runtime, and lower memory usage than method 2. However, method 1 may produce less smooth separate responses and may introduce abrupt changes in the processed data. In contrast, method 2 produced smoother separated responses, although it required more computational resources. Overall, RK3588 again showed better edge-computing performance than RV1126. The runtime of method 1 remained below 0.01 s, while the runtime of the wavelet packet decomposition method remained below 0.25 s. CPU usage was below 1.6%, and most memory usage remained below 1400 KB. It should be noted that the response-separation stage subtracts low-frequency environmental trends but preserves the original data dimensionality and segment size. Therefore, the computational workload passed to the downstream anomaly-detection models remains unchanged. This supports the validity of the subsequent edge-computing performance comparison.

For anomaly-detection algorithms, SVM_lin_ and SVM_RBF_ consistently showed the shortest runtimes across the two field datasets. This indicates that they are suitable for rapid processing in edge-computing environments. In contrast, ARIMAX showed longer runtime, especially as data size increased, making it less suitable for real-time edge deployment. CPU usage remained relatively low for most algorithms, but ARIMAX showed occasional fluctuations. This suggests that ARIMAX may require further optimization for deployment on edge devices. The memory results showed that RNN-based models, including GRU and LSTM, required more memory than the SVM-based models. The SVM-based models maintained low and stable memory usage. This indicates that RNN-based models may provide stronger temporal modeling capability, but they are more resource-intensive. The data-processing throughput proxy further indicates that SVM_lin_ achieves higher processing throughput, which is consistent with its low runtime and efficient processing. Although RNN-based models require more resources, they may still be useful when temporal modeling capability is important and sufficient hardware resources are available.

The inability to deploy some computationally intensive models on the lower-end RV1126 device reflects a practical constraint in real-world edge environments. This limitation shows that software ecosystem compatibility and deployment feasibility are as important as hardware specifications. From a practical deployment perspective, the results suggest different hardware–algorithm matching strategies for SHM edge nodes. For monitoring nodes that mainly perform data cleaning, denoising, and response separation, RV1126-type devices can be used as lightweight preprocessing nodes. When local machine-learning-based anomaly detection is required, RK3588-type devices are more suitable because they provide stronger computing capability and better software–library compatibility. For model selection, SVM-based models are preferable for low-latency and low-memory deployment. LSTM and GRU can be considered when temporal modeling capability is more important and sufficient memory resources are available. Therefore, the choice of edge hardware and algorithm should be guided by the required processing level, latency constraint, memory budget, detection requirement, and software ecosystem compatibility. Recommended SHM deployment choices under different edge-computing constraints are shown in Table 4.

## 5. Conclusions

This study presented a deployment-oriented evaluation of SHM anomaly data-processing algorithms on resource-constrained edge devices. The evaluated workflow included data cleaning, response separation, and anomaly detection. In the data-cleaning stage, cubic spline interpolation was used for missing values, the Laida criterion was used for jump-point and drift corrections, and wavelet threshold denoising was used for noise reduction. In the response-separation stage, temperature-induced and vehicle-induced responses were separated using either the time window and 3σ-based detrending method or wavelet packet decomposition. In the anomaly-detection stage, ARIMAX, SVM_lin_, SVM_RBF_, LSTM, and GRU models were evaluated.

The results showed that the two edge platforms had different deployment characteristics. RK3588 provided stronger computing capability, better software–library compatibility, and better support for machine-learning-based anomaly detection. It was suitable for the full workflow, including preprocessing, response separation, and local anomaly detection. In contrast, RV1126 was more suitable for lightweight preprocessing tasks, such as missing value processing, jump-point and drift corrections, denoising, and response separation. However, several computationally intensive machine-learning models could not be deployed on RV1126 because of its limited system resources and software–library compatibility.

Simulation experiments and field monitoring data from bridge displacement and G2 Highway pavement strain were used to evaluate the algorithms. The evaluation indicators included the runtime, memory usage, CPU usage, and data-processing throughput proxy. The results showed that data-cleaning and response-separation algorithms could be efficiently executed on both edge devices. For anomaly detection, SVM-based models showed lower runtime and memory usage, making them more suitable for low-latency and low-memory edge deployment. RNN-based models required more memory but could be useful when temporal modeling capability is important and sufficient hardware resources are available. These results provide practical evidence for selecting suitable algorithms and edge hardware under different SHM deployment constraints.

Several limitations should also be acknowledged. First, only two edge devices were tested in this study. Although RK3588 and RV1126 represent two typical levels of lightweight industrial edge-computing platforms, they do not cover all available edge hardware architectures. Future work should extend the benchmark to more processors, operating systems, and hardware configurations. Second, the field monitoring datasets did not contain independently verified point-by-point ground-truth anomaly labels. Therefore, the RMSE-based indicators and stability coefficients mainly describe prediction behavior and temporal stability rather than complete classification-level detection effectiveness. Future studies should use expert-annotated datasets or independently verified anomaly records to calculate precision, recall, F1-score, and ROC/AUC. Third, the memory usage reported in this study represents a comparative resident set size difference rather than an absolute peak memory footprint. Continuous peak memory profiling and explicit memory headroom analysis should be included in future deployment-oriented evaluations. Fourth, the current implementation was based on Python and common scientific-computing libraries. The inability to execute the machine-learning models on RV1126 indicates that software ecosystem compatibility and library availability can be as important as raw hardware specifications in edge SHM applications. Future work should further explore low-level embedded implementations, such as C/C++ optimization, lightweight model compression, and knowledge distillation, to improve deployment feasibility on more resource-constrained platforms.

## Figures and Tables

**Table 1:** Edge device processor parameter comparison.

Parameter	Rockchip RK3588	Rockchip RV1126
CPU	8-core (4 × Cortex-A78 @ 2.4 GHz + 4 × Cortex-A55 @ 1.8 GHz)	4-core ARM Cortex-A7 @ 1.5 GHz + RISC-V MCU @ 200 MHz
RAM	8 GB LPDDR4	2 GB DDR3 + 512 KB MCU SRAM
Flash/storage	32 GB eMMC	4 GB eMMC + 1 MB MCU Flash
GPU	ARM Mali-G610 MP4	None
NPU	Supports INT4/INT8/INT16/FP16/BF16/TF32	Supports INT8/INT16
Decoder and encoder	8K H.264/H.265 60FPS	4K H.264/H.265 30FPS
Video input	32MP ISP with HDR and 3DNR	14MP ISP with 3F HDR
Support interface	GIGE/PCIe3.0/PCIe2.0/SATA3.0/ RGMII/TYPE-C/USB3.1/USB2.0	GIGE/SDIO/TDM/ PDM/USB2.0
Supported operating systems	Android and Linux	Linux

**Table 2:** Simulated conditions.

Algorithm Type	Anomaly Condition	Edge Device	Edge-Computing Indices
Missing data processing	2.5%, 5%, and 7.5% missing rates	RK3588 and RV1126	Runtime, CPU usage, memory usage, and data-processing throughput proxy
Jump-point and drift data processing	0.5%, 1.0%, and 1.5% anomaly rates	RK3588 and RV1126	Runtime, CPU usage, memory usage, and data-processing throughput proxy
Denoise processing	Uniform random noise	RK3588 and RV1126	Runtime, CPU usage, memory usage, and data-processing throughput proxy

**Table 3:** Experimental conditions.

Algorithm Type	Monitoring Data Type	Edge Device	Edge-Computing Indices
Data-cleaning algorithm	Displacement and strain data	RK3588 and RV1126	Runtime, CPU usage, memory usage, and data-processing throughput proxy
Response-separation algorithm	Displacement and strain data	RK3588 and RV1126	Runtime, CPU usage, memory usage, and data-processing throughput proxy
Anomaly-detection algorithm	Displacement and strain data	RK3588	Runtime, CPU usage, memory usage, and data-processing throughput proxy

**Table 4:** Recommended deployment choices under different SHM edge-computing constraints.

Deployment Constraint	Recommended Edge Platform	Recommended Algorithm Choice	Practical Implication
Lightweight preprocessing	RV1126 or RK3588	Missing value interpolation, jump-point and drift corrections, and denoising	Suitable for front-end edge nodes with limited computing resources
Low-latency response separation	RV1126 or RK3588	Time-window and 3σ-based detrending method	Suitable for real-time separation of low-frequency environmental trends and vehicle-induced responses
Smoother response separation	RK3588 preferred	Wavelet packet decomposition	Suitable when smoother separated responses are required and additional computation is acceptable
Low-memory anomaly detection	RK3588	SVM_lin_ or SVM_RBF_	Suitable for real-time local anomaly analysis with limited memory consumption
Sequence-based anomaly analysis	RK3588	LSTM or GRU	Suitable when temporal dependencies are important and sufficient computing resources are available
Resource-constrained field node	RV1126	Preprocessing-only or response-separation-only workflow	Suitable for distributed monitoring nodes that mainly reduce data transmission load
Edge node with local intelligence	RK3588	Full workflow, including preprocessing, response separation, and machine-learning anomaly detection	Suitable for bridge, highway, tunnel, and urban infrastructure monitoring requiring local anomaly analysis

## Data Availability

Data are available from the corresponding author upon request.
